# Self-Management and Health Care Use in an Adolescent and Young Adult Medicaid Population With Differing Chronic Illnesses

**DOI:** 10.5888/pcd12.150023

**Published:** 2015-07-02

**Authors:** G. Alexandra Phillips, Nicole Fenton, Sarah Cohen, Karina Javalkar, Maria Ferris

**Affiliations:** Author Affiliations: G. Alexandra Phillips, Sarah Cohen, Karina Javalkar, Maria Ferris, The University of North Carolina, Chapel Hill, Chapel Hill, North Carolina; Nicole Fenton, Dana-Farber/Boston’s Children Cancer and Blood Disorders Center, Boston, Massachusetts.

## Abstract

**Introduction:**

Few studies of adults question the validity of the claim that self-management reduces the use of health care services and, as a result, health care costs. The aim of our study was to determine the relationship between self-management and health care use in a population of adolescent and young adult recipients of North Carolina Medicaid with chronic health conditions, who received care in either the pediatric or adult clinic. Our secondary objective was to characterize the patterns of health care use among this same population.

**Methods:**

One hundred and fifty adolescents or young adults aged 14 to 29 were recruited for this study. Participants completed a demographics questionnaire and the self-management subdomain of the University of North Carolina TRxANSITION Scale. Information on each participant’s emergency department and inpatient use was obtained by using the North Carolina Medicaid Provider Portal.

**Results:**

This cohort had a high level of emergency health care use; average lifetime use was 3.18 (standard deviation [SD], 5.58) emergency department visits, 2.02 (SD, 3.42) inpatient visits, and 12.5 (SD, 23.9 ) days as an inpatient. Age group (pediatric or adult), diagnosis, race/ethnicity, and sex were controlled for in all analyses. Results indicate that patients with a high rate of disease self-management had more emergency department visits and hospitalizations and a longer length of stay in the hospital than did those with a low rate.

**Conclusion:**

In a group of North Carolina Medicaid recipients with chronic conditions, better self-management is associated with more health care use. This is likely the result of many factors, including more interactions with health care professionals, greater ability to recognize the need for emergency medical attention, and the use of the emergency department for primary health care.

## Introduction

As adolescents and young adults with chronic health conditions transition to adulthood, they usually become more responsible for their own care and rely less on their parents or guardians. With proper preparation for and development of self-management skills during the transition from pediatric to adult health care, youths have better health and face fewer negative health outcomes (1,2).

Recently, self-management programs have grown in popularity as studies link better self-management with improved health and lower health care use and costs (3–5). Few studies questioned the validity of the claim that self-management reduces the use of health care services and, as a result, health care costs (6,7). The majority of these studies focused on adult populations, rather than adolescents and young adults who are newly developing self-management skills. The relationship between self-management and use of health care services remains largely understudied in Medicaid populations.

In 2014, 66 million Americans (20% of the US population) had insurance coverage through Medicaid (8). With such a significant amount of the population using federal support for their health insurance, understanding the patterns and underlying factors of health care use by the Medicaid population should lead to more efficient practices and reduce health care costs. In studies of the Medicaid population, few differences exist in economic status, which allows studies to shift their focus to other factors.

The purpose of this study was to determine whether the pattern of self-management and health care use among Medicaid enrollees maintains or contradicts the assertion that better self-management in a broad population of people with chronic diseases results in fewer emergency department visits and hospitalizations. Our secondary objective was to characterize the health care use patterns of young North Carolina Medicaid recipients with chronic conditions. Our findings should be of use to self-management programs and health care practices for adolescents and young adults with chronic illnesses.

## Methods

Participants were selected from Medicaid recipients aged 14 to 29 years with a diagnosed chronic health condition of 6 months or more duration. The diagnoses from this group included chronic kidney disease, end stage renal disease, systemic lupus erythematosus, organ transplant, inflammatory bowel disease, type 1 diabetes, human immunodeficiency virus (HIV), sickle cell anemia, and hypertension. Medicaid recipients were excluded from the study if they had significant cognitive disorders, did not speak English fluently, or if their parent did not speak English fluently. Participants were recruited from both pediatric and adult subspecialties as well as from outpatient clinics and inpatients at The University of North Carolina Hospitals. Recruitment occurred from January 2012 through January 2014 (before The Affordable Care Act was enacted). Informed consent and assent were obtained from parents for participants aged 18 years or younger. The study was approved by the institutional review board at the University of North Carolina at Chapel Hill. Each participant completed the demographics questionnaire, and each was administered the previously published UNC TRxANSITION Scale (9) to measure self-management (for patients in the adult health care setting) or transition readiness (for participants in the pediatric health care setting). Next, each participant’s medical history was reviewed by using the North Carolina Medicaid Provider Portal. The North Carolina Medicaid Provider Portal tracks all medical care for Medicaid recipients in the state of North Carolina. This portal is accessible to registered physicians and other medical professionals. Data on the number of emergency department (ED) visits, hospital admissions, and the length of each of these admissions were collected for 1 year before enrollment and for each patient’s total Medicaid lifetime coverage.

We collected data on age, sex, race/ethnicity, diagnosis, number of medications, and age at diagnosis from each participant. Participants were grouped into 2 groups, adult and pediatric, on the basis of the clinic in which the patient received health care. The percentage of the participant’s life with disease was calculated by dividing the age at diagnosis by the participant’s current age.

Disease self-management or transition-readiness was assessed by using the UNC TRxANSITION Scale (9). This 33-question semi-structured interview was administered by a health care provider and assessed disease self-efficacy, that is, what the people interviewed believed they could do to manage their illness and their knowledge of their disease. The UNC TRxANSITION Scale consists of 10 subdomains, among them, patients’ knowledge about their medications, type of illness, nutrition, insurance, reproductive health, disease self-management, and self-activation. The disease self-management subdomain, which was the focus of this study, asks questions such as, “Do you usually remember to take medicines on your own?” or, “Do you usually make your own doctor appointments?” On the basis of the participant’s response, the health care provider determines a score: no is scored as a 0, yes as a 1, and sometimes as 0.5. There is an N/not applicable option available if a question does not pertain to the participant. If, for example, a participant does not take any medications, the questions pertaining to medication management will have no bearing on the total self-management score. The score for each response is then averaged to obtain the final subdomain score on a scale of 0 to 1, with 0 being no knowledge, 0.5 some knowledge, and 1 being total knowledge of skills. The generic nature of the questions has allowed the use of the UNC TRxANSITION Scale to measure self-management and transition readiness in adolescents and young adults with various chronic diseases. This tool has been found to have satisfactory content and construct validity (9).

Data on the participant’s use of health care services was obtained from the North Carolina Medicaid Provider Portal. The following variables were collected: ED visits in the previous year and over the course of the patient’s Medicaid lifetime coverage, number of inpatient admissions in the previous year and over the course of the patient’s Medicaid lifetime coverage, and the length of hospital admissions (both in the previous year and over the course of the patient’s Medicaid lifetime coverage).

We conducted linear regressions by using the following independent variables: self-management score, age group (pediatric or adult), diagnosis, race/ethnicity, and sex. For each regression the outcome was the number of ED visits, the number of inpatient visits, or the number of days spent as an inpatient. These regressions were calculated for history of health care use in the previous year and lifetime use. Significance was set with a *P* value of <.05.

## Results

We enrolled 150 adolescents and young adults with chronic health conditions in the study (Table 1), 55.0% from a pediatric clinic or inpatient service and 45.0% from an adult clinic or inpatient service. The majority of participants were female (59.3%) and African American (63.3.%), with the most common diagnoses being systemic lupus erythematosus (19.3%) and organ transplant (18.7%). The mean age of participants was 19.6 years (standard deviation [SD], 3.6), and the mean age at diagnosis of the chronic health condition was 9.8 years (SD, 6.5). Of the 150 participants, 40.0% were prescribed medications. The participants had a median of 0 (IQR, 0–1.0) ED visits in the previous year and 1.0 (IQR, 0–4.0) inpatient visits in the past year. Participants had a median of 0 (IQR, 0–13.0) days inpatient over their Medicaid lifetime coverage (Table 2). 

**Table 1:** Participant (N = 150) Characteristics, Study of Adolescent and Young Adult Medicaid Recipients Aged 14 to 29 Years, Chapel Hill, North Carolina, 2014

Characteristic	N (%)^a^
**Race/ethnicity**	
African American	95 (63.3)
White	32 (21.3)
Other	10 (6.7)
Hispanic or Latino	7 (4.7)
American Indian	5 (3.3)
Asian	1 (0.7)
**Sex**	
Female	89 (59.3)
Male	61 (40.7)
**Age, mean (SD), y**	19.6 (3.6)
**Age at diagnosis, median (interquartile range), y**	11.5 (14.0-4.0)
**Diagnosis **	
Systemic lupus erythematosus	29 (19.3)
Organ transplant	28 (18.7)
Inflammatory bowel disease	23 (15.3)
Chronic kidney disease	20 (13.3)
Other kidney disease	15 (10)
Sickle cell anemia	15 (10)
Type 1 diabetes	7 (4.7)
Hypertension	6 (4.0)
End-stage renal disease	4 (2.7)
Human immunodeficiency virus	3 (2.0)
**Clinic **	
Pediatric patients	83 (55.0)
Adult patients	67 (45.0)

Abbreviation: SD, standard deviation.

a Values are n (%) unless otherwise indicated.

**Table 2:** Average Health Care Use and Self-Management, by Diagnosis, Study of Adolescent and Young Adult Medicaid Recipients Aged 14 to 29 Years, Chapel Hill, North Carolina, 2014^a^

Diagnosis	ED Visits, 1 Year Prior^a^	ED Visits, Lifetime^a^	Inpatient Visits, 1 Year Prior	Inpatient Visits, Lifetime	Total Number of Days Inpatient, 1 Year Prior	Total Number of Days Inpatient, Lifetime	Self Manage-ment Score^b^
	Median (Interquartile Range)						
Chronic kidney disease	0	0 (0–1.0)	0	0 (0–0.8)	0	0 (0–2.0)	0.38 (0.3–0.5)
Other kidney disease	0 (0–1.0)	1.0 (0–2.0)	0	0 (0–2.0)	0	0 (0–7.0)	0.33 (0.3–0.8)
Systemic lupus erythematosus	1.0 (0–3.5)	4.0 (1.0–11.5)	0 (0–2.0)	2.0 (0–6.0)	0 (0–12.0)	8.0 (0–26.5)	0.58 (0.4–0.8)
Inflammatory bowel disease	0 (0–1.0)	0 (0–4.0)	0 (0–3.0)	0 (0–1.0)	0 (0–1.0)	0 (0–12.0)	0.40 (0.3–0.7)
Sickle cell anemia	0 (0–1.0)	0 (0–4.0)	0 (0–1.0)	0 (0–2.0)	0 (0–3.0)	0 (0–10.0)	0.33 (0.2–0.6)
Type 1 diabetes	0	0	0	0	0	0	0.43 (0.4–0.50)
Hypertension	0	0(0–0.25)	0	0	0	0	0.46 (0.15–0.6)
Organ transplant	0 (0–0.8)	2.0 (0–5.0)	0 (0–1.0)	3.0 (0.3–5.0)	0 (0–6.5)	12.0 (0–31.0)	0.5 (0.3–0.9)
End-stage renal disease	6.5 (2.0–8.0)	13.0 (3.3–22.0)	6.0 (2.0–7.0)	9.0 (3.0–10.5)	84 (18.3–114.5)	86.5 (18.5–118.5)	0.5 (0.4–0.7)
HIV	0 (0–1.0)	1.0 (0–7.0)	0(0–1.0)	0 (0–1.0)	0	0 (0–18.0)	0.4 (0–0.8)
Total	0 (0–1.0)	1.0 (0–4.0)	0 (0–1.0)	0 (0–3.0)	0 (0–4.0)	0 (0–13.0)	0.5 (0.3–0.7)

Abbreviations: ED, emergency department; HIV, human immunodeficiency virus.

a Values for 1 year prior are from data collected for the participant’s history in the year before enrollment in the study. Lifetime values are for the entire participant history available in the North Carolina Medicaid Provider Portal.

b The self-management score is a total proportion of performance on a scale of 0 to 1. A score of 0 represents no knowledge, and a score of 1 represents total knowledge of skills.

Self-management was a significant positive predictor of health care use when controlling for age group (pediatric or adult), participant’s diagnosis, race/ethnicity, and sex (Table 3). Those with greater self-management skills spent a greater number of days as an inpatient (β = 0.25, *P* = .003) and had a greater number of inpatient visits over their lifetime (β = 0.18, *P* = .04). Self-management was also a significant positive predictor of ED visits both in the participant’s Medicaid lifetime coverage (β = 0.17, *P* = .05) and in the year before the study (β = 0.19, *P* = .03). Therefore, participants with greater self-management skills had greater numbers of ED visits than those with lower self-management skills.

**Table 3:** Linear Regression Models for Health Care Use and Disease Self-Management, With Controls, Chapel Hill, North Carolina, 2014

Outcome	Independent Variable	β	*P* a
Number of days inpatient (lifetime)	Self-management score	0.253	.003
	Clinic (pediatric or adult)^b^	−0.208	.02
	Diagnosis	0.223	.007
	Race/ethnicity	0.002	.98
	Sex	0.038	.64
Number of inpatient visits (lifetime)	Self-management score	0.176	.04
	Clinic (pediatric or adult) b	−0.164	.06
	Diagnosis	0.124	.14
	Race/ethnicity	0.062	.46
	Sex	−0.056	.51
Number of emergency department visits (lifetime)	Self-management score	0.171	.05
	Clinic (pediatric or adult) b	−0.102	.25
	Diagnosis	0.053	.53
	Race/ethnicity	0.001	.99
	Sex	0.048	.57
Number of emergency department visits (1 year prior)	Self-management score	0.191	.03
	Clinic (pediatric or adult)^b^	−0.150	.09
	Diagnosis	−0.021	.80
	Race/ethnicity	0.014	.87
	Sex	0.008	.93

a *P* values calculated with linear regression analysis.

b Clinic where study participant received health care.

Additionally, 2 of the control variables, diagnosis and clinic where participant received health care (pediatric or adult), were significant predictors of the lifetime number of days of inpatient health care. Specifically, pediatric patients had more lifetime days inpatient than did adult patients (β= −0.21, *P* = .02). Also, patients with end stage renal disease and an organ transplant had significantly more lifetime days inpatient (β = 0.22, *P* = .007) than did patients with the other illnesses examined.

To account for the wide age range, a *t* test was performed to compare self-management scores and health care use by pediatric and adult participants. Self-management scores differed significantly for pediatric and adult groups [*t*_148_ = −3.36, *P* = .001] and for the length of stay as an inpatient in the year prior to this study [*t*_105_ = 2.14, *P* = .04], such that pediatric patients had lower self-management skills and longer inpatient stays. 

## Discussion

Few studies to date have focused on adolescents and young adults receiving Medicaid, and none have focused on a sample with such significant disease severity. Compared with the study conducted by Shatin and colleagues (10), one of the largest studies conducted to date that examines the health care use of pediatric Medicaid patients with a chronic illness, the health care use of our sample was significantly higher than would have been expected (10). Our sample had both more ED visits and hospital admissions than previous studies have predicted. This upward shift may suggest that increases in the availability of resources to improve self-management for adolescents and young adults facilitates more knowledge of their condition and helps them know when to seek out medical care. It may also suggest that this sample is more ill than samples in other published studies. This finding may have multiple reasons, one of which is the illness populations included in this study have greater disease severity and progression than those of prior studies (ie, organ transplantation and end stage renal disease vs asthma or attention deficit–hyperactivity disorder). Disease severity may be related to the difficulties these patients experience in finding primary care providers who are comfortable with complex medical conditions. Participants with more severe diagnoses, such as systemic lupus erythematosus or end-stage renal disease, used health care services more than participants with less severe conditions, such as diabetes or hypertension. The group with more severe illnesses also had higher self-management scores (Table 2). Given the significant use of health care services by our study sample, an understanding of factors that may reduce ED visits and inpatient admissions is needed.

One factor that has been proposed to help prevent excessive ED and inpatient health care use is disease self-management. Our study found that when patients had higher levels of disease self-management, they used health care more. This may be related to patients’ having more interactions with the medical system (ie, their disease self-management and knowledge increases because of the additional opportunities to hear health-related information). Alternatively, it may be that when patients have high levels of self-management, they know when they are medically at risk. Depending on their age, they may seek out medical care of their own accord or inform their parents or caregivers of sudden changes in symptoms or health. The patients then go to the ED where they are admitted for further care. A final interpretation is that patients with Medicaid are using the ED as their primary care provider and therefore their ED use is high regardless of their disease self-management. In our sample, 80.9% of the participants had a primary care provider. Patients without a primary care provider had more ED visits and longer stays in the hospital over their lifetime Medicaid coverage history than those with a primary care provider. Our data did not include the number of out-patient visits. Regardless of how well or poorly people manage their chronic illness, if they have urgent need of health care, those without a regular primary care provider will obtain health care in an ED. This may suggest the importance of interventions aimed to reduce use of the ED for care that could be managed by a primary care provider (11). Longitudinal investigations will allow for a greater understanding of the mechanisms that are underlying the relationship between health care use and disease self-management.

Our findings contradict the studies that examined the association between disease self-management and health care use. According to studies of adults, better self-management leads to better self-care and fewer negative health outcomes, which reduces hospitalizations and ED use (12–14). Other studies showed that the relationship between self-management and health care use is inconclusive (15,16). These studies generally had older participants than those in our study. A recent systematic review that concluded that self-management interventions reduce health care use included 148 studies in its analysis but excluded studies with participants under the age of 18 (17). In contrast, our study included adolescents and young adults aged 14 to 29 years with a mean age of 20. A young patient population is newly learning self-management skills as they transition from reliance on parents and guardians to self-care, which may influence their use of health care services. The comparison of self-management performance in our sample showed that participants from the adult clinic had a higher mean score (0.57) than the pediatric group’s mean score (0.42). This could explain the significant negative relationship between health care use and clinic where participants received health care (Table 3), showing that younger patients spend more days in the hospital. However, this significant result was only found when looking at the number of days spent as an inpatient, which is determined by the health care provider and is largely out of the patient’s control. There may be differences in practice between pediatric and adult health care providers that could explain this result if providers of pediatric care tend to keep their patients in the hospital longer than providers of adult care for the same condition. This may be determined by future research.

This study had limitations. The study was a cross-sectional examination of the relationship between disease self-management and health care use. The study used only 1 measure of disease self-management, which assessed only patients’ perceptions of their own self-management rather than a health care provider’s perception, and recruitment occurred at only 1 location. Although the University of North Carolina TRxANSITION Scale is administered by a health care provider, the results could be biased by patients’ reports of their own self-management. A strength of the study is that it incorporated unique components. It is the first study to focus on adolescents and young adults with chronic illness in the Medicaid populations. Because these youths may have received health care in both the pediatric and adult clinics, we recruited from both clinics. Another strength of our study, is that the provider portal gave us data on use of health care services and eliminated the biases that can occur from self-reported patient data. Additionally, this study focused on several chronic illnesses and included a representative sample of patients from the North Carolina Medicaid population served at University of North Carolina Hospitals. Furthermore, no research investigated the effect of self-management on health care use by a Medicaid-only population. More than 1 in 5 Americans were covered by Medicaid at some point in 2014 (8), so their behavior and health care practices could have profound effects on national health care costs. Studies of the Medicaid population are important for future research directed at lowering health care costs.
